# Identification of a Novel Polerovirus in Cocoa (*Theobroma cacao*) Germplasm and Development of Molecular Methods for Use in Diagnostics

**DOI:** 10.3390/pathogens12111284

**Published:** 2023-10-26

**Authors:** Ihsan Ullah, Muhammad Kamran, Jim M. Dunwell

**Affiliations:** 1 School of Agriculture, Policy and Development, University of Reading, Reading RG6 6EU, UK; i.ullah@reading.ac.uk; 2 Plant Pathology Research Institute, Ayub Agricultural Research Institute, Faisalabad 38850, Pakistan; mkamran.uaf.pk@gmail.com

**Keywords:** cacao, polerovirus, genome, RT-PCR, NGS, ORF

## Abstract

The cocoa crop (*Theobroma cacao* L.) is known to be a host for several badnaviruses, some of which cause severe disease, while others are asymptomatic. Recently, the first preliminary evidence has been published concerning the occurrence of a polerovirus in cacao. We report here the first near-complete genome sequence of cacao polerovirus (CaPV) by combining bioinformatic searches of cacao transcript databases, with cloning from the infected germplasm. The reported novel genome has all the genome features known for poleroviruses from other species. Pairwise identity analyses of RNA-dependent RNA polymerase and coat protein indicates < 60% similarity of CaPV with any reported poleroviruses; hence, we propose that the polerovirus isolate reported in this study is a novel polerovirus. The genome sequence information was also used to develop a multiplex RT-PCR assay, which was applied to screen a selected range of germplasms and to identify several infected clones. Although there is no evidence that this virus causes any severe disease, this new information, together with a robust diagnostic assay, are of strategic importance in developing protocols for the safe international transfer of cacao germplasms.

## 1. Introduction

Cacao (*Theobroma cacao*) holds significant economic importance for both producing and consuming countries. This tropical evergreen tree is predominantly grown in West Africa, Central and South America, and Southeast Asia. The cacao beans, also referred to as cocoa, represent an important ingredient for food, pharmaceutical and cosmetics industries. In 2022, the global chocolate confectionery market alone generated a revenue of approximately 0.22 trillion U.S. dollars [1]. The sustainable production of cocoa is facing numerous challenges. Lack of access to improved planting materials, poor agricultural practices, impoverished soils and nutrient deficiencies, climate change and losses due to pests and diseases influence cacao productivity.

Fungal and other diseases like black pod rot, caused by the *Phytophthora* species, and witches’ broom of cacao, caused by *Moniliophthora perniciosa*, cause significant loss in cacao yields. Similar to other crop species, cacao is also susceptible to viral infections. Until recently, most viral diseases of cacao were believed to be caused by the members of the genus *Badnavirus*. On the basis of their impact on plant growth and productivity, cacao badnaviral diseases can be categorized into two groups. The first, cacao swollen shoot virus disease (CSSVD), is a serious threat to cacao trees. CSSVD is caused by seven species related to the genus *Badnavirus* [2] and is confined to the cacao growing region of West Africa, which contributes over 74% of global cacao production [3]. The second group of badnaviral diseases are caused by the species *cacao bacilliform Sri Lanka virus* (CBSLV), *cacao mild mosaic virus* (CaMMV) and *cacao yellow vein banding virus* (CYVBV). The latter two species have been reported to infect cacao plantations in multiple countries [4,5,6,7,8], whereas CBSLV has been detected in Sri Lanka [2]. To date, none of these infections have been associated with any effect on plant growth or productivity. Badnaviruses have also been reported as endogenized in the cacao genome [9,10].

*Solemoviridae* is a family of plant infecting viruses that can significantly influence yield and quality in crops such as cotton [11]. The family consists of four genera: *Enamovirus, Polemovirus*, *Polerovirus* and *Sobemovirus* [12]. The 4–6 kb non-enveloped, positive-strand RNA genome comprises 4–10 open reading frames (ORFs). The genome contains a virus protein genome-linked (VPg) cap at the 5′ terminus, whereas the 3′ terminus is non-polyadenylated but is protected by a stable stem-loop or tRNA-like structure [13]. The members of these four genera vary regarding nucleotide sequence, genome size and number and arrangement of ORFs. Poleroviruses are closer to the enamoviruses in terms of the structure and expression of the genome [14]. Enamoviruses and poleroviruses contain a non-coding intergenic region, which is absent in the genomes of polemoviruses and sobemoviruses. Enamoviruses and poleroviruses vary in terms of nucleotide sequence and coding regions, with a major difference being the absence of the movement protein in enamoviruses [15].

Recently, two studies have reported the detection of virus species from the genus *Polerovirus* in cacao trees in the Americas. The first study detected polerovirus-like partial sequences, designated as cacao leafroll virus, in cacao germplasms imported into USDA-ARS-SHRS cacao quarantine facilities. The plants exhibited leaf distortion with downward rolling of leaves, discoloration and yellow speckling or mottling [16]. The second study reported the infection of cotton leafroll dwarf virus (CLRDV) in cacao trees in Bahia, Brazil, exhibiting virus-like symptoms such as leaf mosaicism, vein clearing and yellow spots [17]. Illumina sequencing and RT-PCR of the cacao leaf samples yielded two highly similar contigs, which represented ~29% of the CLRDV genome. The contigs showed >85% identity with CLRDV isolates previously reported in South America and the USA. Interestingly, 14 out of 30 symptomatic plants tested negative. It was argued that the symptoms on the negative plants could have been caused by other viral groups or abiotic stress.

In terms of host range, Polerovirus is a diverse genus infecting a wide range of plants, from monocots to eudicots. Poleroviruses are phloem-limited viruses, which are believed, under usual conditions, to be solely transmitted by aphids. The infection may cause stunt, interveinal yellowing, discoloration of the main leaf vein, leaf curling and leaf necrosis. The International Committee on Taxonomy of Viruses (ICTV) at present has recognised 26 member species in the genus. Six hundred and two complete genome sequences for species have been recognised by ICTV, as well as unrecognised poleroviruses that have been reported in NCBI (https://www.ncbi.nlm.nih.gov/nuccore; accessed on 20 September 2023), out of which 42 genomes have been recognised as reference sequences. These reported genomes are ascribed to over 90 host plant species. The poleroviruses genome length in 26 exemplar species listed in the Virus Metadata Resource (VMR; https://ictv.global/vmr; last accessed on 30 September 2023) ranged from 5612 (maize yellow dwarf virus-RMV; NC_021484) to 6244 nucleotides (NC_075422; pepper vein yellows virus).

The polerovirus genome consists of six major ORFs. A non-conserved 5′-proximal ORF0 encodes an RNA silencing suppressor [18]. The ORF1 encodes a polyprotein P1, a protease- and genome-linked protein. The Polerovirus genome encodes the RNA-dependent RNA polymerase (RdRP) by −1 frameshift (−1 rfs) translational fusion of ORF1 through ORF2. The ORF2 is translated from genomic RNA using a ribosomal leaky scanning strategy [19]. The −1 rfs signal of the polerovirus consists of the slippery sequence 5′-GGGAAAC-3′ followed by a putative small pseudoknot. Other slippery heptanucleotide variants can also be found in polerovirus genomes [20,21]. The 3′-proximal ORFs are separated from 5′-proximal ORFs by a non-coding intergenic region. These ORFs are translated from sub-genomic RNA(s). The ORF3 and ORF4 encode the coat protein (CP), and movement protein (MP). The ORF4 overlaps ORF3 but in a different reading frame. The ORF3 and ORF5 combine to form the coat protein-readthrough domain fusion protein (P3-P5). In the process, ribosomes skip the stop codon at the end of ORF3 and continue to translate ORF5 in the same frame [22]. A short non-AUG-initiated ORF3a, which encodes a different MP (P3a), has also been described in poleroviruses [23].

Though polerovirus infections have been found in cacao, and though partial nucleotide sequences of the RdRP gene, along with CP and MP genes, have been reported, the genome of cacao polerovirus has not been sequenced. Here, we report the first near-complete sequence of a novel polerovirus with the proposed name of cacao polerovirus (CaPV). We also report a multiplex diagnostic RT-PCR test for the detection of this virus.

## 2. Materials and Methods

### 2.1. Analysis of Transcriptome Datasets

The Sequence Read Archive (SRA, https://www.ncbi.nlm.nih.gov/sra; last accessed on 30 June 2023) was searched for publicly available RNA sequence (RNA-Seq) datasets of *T. cacao*. Initially, the datasets were searched with the BLASTn tool for cacao polerovirus, using a reference database consisting of five cacao leafroll virus sequences (GenBank accessions ON745771-75). The raw read FastQ format files of the RNA-Seq dataset in BioProject PRJNA558793 were downloaded from the SRA for comprehensive analysis. The OmicsBox 3.0.30 software package (available at https://www.biobam.com/omicsbox; last accessed on 30 September 2023) was employed, to align the read to the reference databases using a BWA mapper. The Trinity assembler software 2.14.0 in the OmicsBox was used to construct an RNA-Seq de novo transcriptome assembly from the RNA-Seq dataset of cacao accession IMC60 (SRR9938230). A BLASTx search was conducted in the functional analysis module of the OmicsBox, using the de novo transcriptome assembly as a query against the non-redundant protein database (nr) of the *Solemoviridae* family.

### 2.2. Sequence Analysis and Phylogeny

The de novo contig identified in the BLASTx search, with enquiry set to Solemoviruses, was subjected to sequence analysis. The putative ORFs and predicted amino acid sequences were identified using the CLC sequence viewer (Qiagen, Manchester, UK). The protein domain features were predicted using the InterPro database (https://www.ebi.ac.uk/interpro/; last accessed on 30 September 2023). The amino acid sequences of the RdRP and CP of 43 representative species in *Solemoviridae* family were downloaded from NCBI (http://www.ncbi.nlm.nih.gov/; last accessed on 30 September 2023). The obtained sequences were used as the source data for pairwise comparisons, using Sequence Demarcation Tool version 1.2 (SDT v1.2) [24], and the construction of phylogenetic trees using the maximum likelihood method in the RAxML, with a JTT substitution model [25]. One thousand bootstrap samplings were carried out. Trees were drawn in MEGA11 [26].

### 2.3. RNA Extraction and cDNA Synthesis

A fully expanded young leaf was sampled for RNA extraction from each of 26 cacao accessions from the germplasm held at International Cocoa Quarantine Centre, Reading, UK (ICQC-R). The selected germplasm was imported from the International Cacao Collection (IC3) at Centro Agronómico Tropical de Investigación y Enseñanza (CATIE), Costa Rica, between 1988 and 2021.

Four leaf discs of 8 mm diameter were ground with liquid nitrogen in a 2 mL Eppendorf tube in the presence of ceramic beads, using a TissueLyser II (Qiagen, UK). Total RNA was isolated by a modified CTAB method, followed by purification with the Spectrum^TM^ plant total RNA kit (Merck, Gillingham, UK) according to the manufacturer’s instructions. DNA digestion was performed with an On-Column DNase I Digestion Set (Merck, Gillingham, UK) for the removal of trace amounts of genomic DNA. The quality and quantity of isolated RNA was determined using a NanoDrop 2000 Spectrophotometer (Thermo Fisher Scientific, Horsham, UK). Aliquots of RNA were also run on 1.2% agarose gel for quality analysis. Isolated total RNA (300–1000 ng) was transcribed into cDNA using the SuperScript™ IV VILO™ Master Mix with ezDNase™ Enzyme (Thermo Fisher Scientific, UK). The cDNA was diluted 5-fold with RNase- and DNase-free water.

### 2.4. Primer Design and RT-PCR Amplification

The primer sets were designed with the online tool Primer3Plus (https://www.bioinformatics.nl/cgi-bin/primer3plus/primer3plus.cgi; last accessed on 30 September 2023). The cacao polerovirus-specific diagnostic primers were designed from ORFs encoding RdRP, MP and P5 proteins in the identified contig. The *T. cacao* acyl carrier protein 1 gene (ACP1; LOC18599903) was selected as the internal control (IC), as it constitutively expresses across the various cacao tissues [27]. A primer set was designed from exon two and three of the APC1 gene to be used as the IC in diagnostic multiplex RT-PCR assays. Primer sets were designed from the 5 and 3′ UTR region for the amplification of a near-complete genome of CaPV.

For the diagnostic analysis, a 20 μL single or multiplex RT-PCR reaction consisting of 10 μL of Platinum Hot Start PCR Master Mix, 1.5 μL each of 5 μM primers, 5 μL of cDNA template and x μL of PCR water was performed, with thermal cycling of one cycle of 94 °C for 2 min, followed by thirty five cycles of 94 °C for 10 s, 60 °C for 10 s and 68 °C for 25 s.

The Phusion Flash PCR Master Mix was used to amplify the genome sequence which was harbouring coding sequences. The RT-PCR reaction, which contained 12.5 μL of PCR Mix, 2 μL each of 5 μM forward and reverse primers, 5 μL of cDNA template and 4.5 μL of PCR water, was run at one cycle of 98 °C for 10 s, followed by thirty five cycles of 98 °C for 1 s, 60 °C for 5 s and 72 °C for 150 s. Final extension was performed at 72 °C for 3 min. The RT-PCR reactions were performed in a thermal cycler (Veriti, Applied Biosystems). The amplicon was cloned with a Zero Blunt™ TOPO™ PCR Cloning Kit for Sequencing. The cloned RT-PCR products were sequenced using Sanger technology (Source Bioscience, Nottingham, UK). The sequencing data were assembled using SeqMan Ultra contig assembly software Version 17.3 (DNASTAR, Madison, USA). The RT-PCR products were resolved on 1–1.7% agarose gel and stained with ethidium bromide. Information about the primers used in this study, including primer sequences, specific targets, coordinates and product size, is described in Table 1.

## 3. Results

### 3.1. Detection of Polerovirus Sequences in Transcriptome Datasets

A recent study on the detection of partial sequences of a polerovirus (designated as cacao leafroll virus) in an imported cacao germplasm at the USDA-ARS-SHRS cacao quarantine facilities [16] led us to screen the available next generation sequencing (NGS) data in public data depositories against the reported sequences. The SRA database search of *T. cacao* RNA-Seq data found 954 datasets, generated from 100 cacao accessions in 18 BioProject studies (Table S1). The BLASTn search for the reported partial sequences identified 33 datasets of 11 cacao accessions in BioProject PRJNA558793 [28], with a considerable number of reads similar to the query sequences. This particular BioProject was conducted on 40 cacao accessions and contains 276 cacao RNA-Seq datasets. Multiple sets of RNA-Seq data were produced for 31 accessions, representing four cacao genetic groups. One set comprised the data produced from leaf samples collected from these 31 accessions from the IC3 at CATIE. Cuttings from these trees were imported and vegetatively propagated at The Pennsylvania State University (PSU), USA, in 2020. Subsequently, mature leaves were sampled and subjected to RNA-Seq, to generate another set of transcriptome data [29]. The data from 22 runs, representing positive datasets for 11 accessions at both locations, were downloaded and analysed. Information on accession name, genetic group, geolocation, run number, data size, number of mapped reads and coverage depth is provided in Table 2. Although the number of mapped reads and coverage depth varied considerably among the cacao accessions, similar patterns were observed at both locations. Coverage depth ranged from 2.4 to 14.1-fold and from 0.8 to 13.8-fold when considering the data of 11 cacao accessions, each from CATIE and PSU, respectively. The data for the number of mapped reads varied between the locations, primarily due to differences in sequencing depth. Based on the prevalence of the mapped reads, accession IMC60 had the maximum amount of virus titre, followed by PA 16. The accessions SPEC 54/1 and AMAZ 12 had a low amount of virus.

### 3.2. Discovery of Novel Cacao Polerovirus Genome

The detection of a considerable number of cacao leafroll virus sequence reads in the RNA-Seq data of multiple cacao accessions triggered a search for the complete genome of the virus. The sequencing dataset SRR9938230, which showed the maximum number of virus reads, was used to construct a de novo assembly. This specific dataset, comprising 12.1 M paired-end reads, was generated from a leaf sample library of cocoa accession IMC60 from CATIE. The BLASTx searches of the de novo assembled contigs against the NCBI RefSeq protein database of the *Solemoviridae* family found a contig of 5942 nucleotides (nt), similar to members of the genus *Polerovirus*. A total of 820 reads were mapped to the identified contig, covering the entire sequence. Multiple sequence alignment of the contig with the previously reported 1439 bp cacao leafroll virus sequences (ON745771–ON745775) found >99.2% similarity (Figure S1). A BLASTn search of the nucleotide sequence of the contig against the RefSeq genome database revealed 69% identity (score: 553; E-value: 1 × 10^−154^; query coverage: 28%) with the potato leaf roll virus (NC_001747), a member of the *Polerovirus* genus in the *Solemoviridae* family. We therefore regarded this contig to be a near-complete genome of a novel species of the genus *Polerovirus* and propose to name it cacao polerovirus (CaPV; GenBank accession number OR605721).

#### 3.2.1. Genome Organization of CaPV

**Figure 1:**
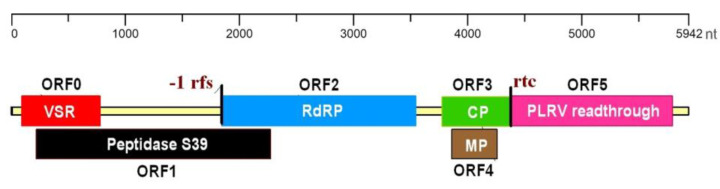
A schematic representation of the genome organisation of the cacao polerovirus (CaPV). Horizontal bars represent the arrangement of the predicted open reading frames (ORFs). The figure is drawn to scale. The bars are labelled with the ORF numbers and the conserved family/domain predicted in the gene products, which include P0 suppressor of silencing (VSR), peptidase S39, RNA-directed RNA polymerase (RdRP), coat protein (CP), movement protein (MP) and potato leaf roll virus readthrough protein (PLRV readthrough). Vertical lines indicate the positions of the predicted −1 ribosomal frameshift (−1 rfs) and predicted readthrough stop codon (rtc). nt denotes nucleotide.

The NGS-derived near-complete genome of CaPV possesses the typical genome organization of previously characterized poleroviruses. It contains six ORFs, flanked by 57 and 137 nt long 5′ and 3′ untranslated regions (UTRs), respectively. The ORFs 0, −1 and −2 are separated from ORFs 3, −4 and −5 by a 223 nt non-coding intergenic region. The FSFinder2 webtool (http://wilab.inha.ac.kr/fsfinder2/; last accessed on 30 September 2023) predicted the slippery heptanucleotide (5′-GGGAAAA-3′) at nt 1845. A linearized representation of the genome organization is provided in Figure 1. The 687 nt long predicted ORF0 (nt 90–776) encodes a protein (P0) of 26.7 kDa. The P0 (IPR006755) is a suppressor of RNA-mediated gene silencing [17]. The predicted ORF1 is found to be 2058 nt in length (nt 217–2274) and encodes the P1 protein. This 76.2 kDa protein contains a peptidase S39b domain (IPR000382), which is putatively involved in the cleavage of the polyprotein. The CaPV genome encodes the RNA-dependent RNA polymerase (RdRP; IPR001795) of 125.4 kDa by −1 frameshift translational fusion of ORF1 (nt 217–1845) through ORF2 (1845–3551 nt). The 603 nt long ORF3 (nt 3778–4380) and 402 nt long ORF4 (nt 3863–4264) encode the P3 protein (CP; IPR001517) of 22.2 kDa and P4 protein (MP; IPR001964) of 15.3 kDa, respectively. The ORF4 is initiated by a CTG start codon and overlaps ORF3, but in a different reading frame. The ORF5 (4384–5802 nt) encodes the readthrough protein (PLRV readthrough domain; IPR002929), which is translated via a readthrough of the termination codon of ORF3, and hence allows the transcription of ORF5 to translate an extended coat protein.

#### 3.2.2. Similarity Analyses of CaPV and Selected Solemovirids

The amino acid sequences of CP and RdRP proteins of 43 selected species in the *Solemoviridae* family, listed as exemplar species in the Virus Metadata Resource by the ICTV, were downloaded and compared with the respective proteins in CaPV. It includes twenty two, eighteen, two and one species of the genera *Polerovirus*, *Sobemovirus*, *Enamovirus* and *Polemovirus*, respectively.

The pairwise comparison of CP revealed 40–57% similarity of CaPV to the selected polerovirus species and showed the highest sequence similarity with cotton leafroll dwarf virus (YP_003915151) and melon aphid borne yellows (YP_001949873). The degree of identity of the selected enamoviruses and sobemoviruses to CaPV ranged from 32 to 38% and 18 to 29%, respectively. The CaPV was found to be 25% similar to *Poinsettia latent virus* (PnLV), a Polemovirus (Table S2).

The pairwise comparison, based on the amino acid sequence of the RdRP protein of CaPV, also showed high similarity with poleroviruses, with a range of 43–58%. The selected sobemoviruses and enamoviruses were found to be 28–39% and 33–34% similar to CaPV, respectively (Table S3). The sequence of RdRP protein encoded by CaPV was found to be 62% similar to that of PnLV, which is an expected result, as PnLV was found to be a chimeric virus with a closer relationship of its RdRP genes and CP genes to those of poleroviruses and sobemoviruses, respectively [30].

The genome organization and pairwise identity of CP and RdRP proteins indicate that CaPV is a member of the genus *Polerovirus*. The ICTV species demarcation criterion for the genus *Polerovirus* is based on >10% differences in the amino acid sequence identity of any gene product [31]. Pairwise identity analyses of CP and RdRP proteins indicate < 60% similarity of CaPV with any reported poleroviruses; hence, we propose that the polerovirus isolate reported in this study is a novel polerovirus.

#### 3.2.3. Phylogenetic Analysis

**Figure 2:**
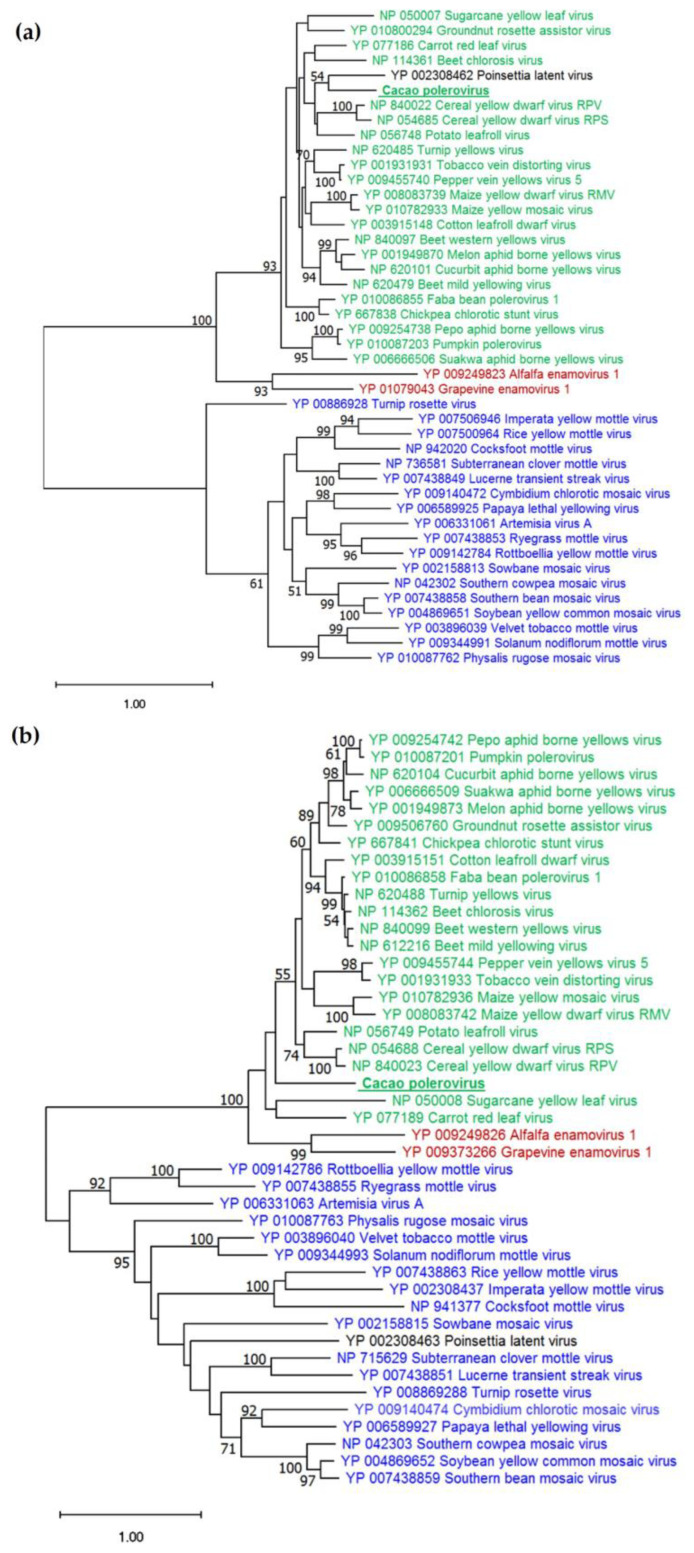
Phylogenetic analysis of selected virus species from four genera of the *Solemoviridae* family, based on amino acid sequences of coat protein (**a**) and RNA-directed RNA polymerase (**b**), using the maximum likelihood method. The numbers near the branches represent ultrafast bootstrap values, derived from 1000 replications (values ≥ 50 are shown). Green, black, red and blue font colours denote species related to the genera *Polerovirus*, *Polemovirus*, *Enamovirus* and *Sobemovirus*, respectively. The underlined label in bold font represents the cacao isolate discovered in this study.

Amino acid sequences of CP or RdRP proteins, of the CaPV isolate discovered in this study and the 43 species mentioned above, were used to construct phylogenetic trees, using the maximum likelihood method in the RAxML with a JTT substitution model. The tree, constructed with RdRP sequences, separated 44 sequences into three distinct clades, representing the *Polerovirus, Enamovirus*, and *Sobemovirus* genera, with >90% bootstrap support (Figure 2a). The CaPV isolate was grouped with the genus *Polerovirus*. A similar pattern was recorded in the tree constructed with CP sequences (Figure 2b). As expected in these phylogenetic comparisons, Poinsettia latent virus, from the genus *Polemovirus*, was grouped with the genera *Polerovirus* and *Sobemovirus* in CP- and RdRP-based trees, respectively.

### 3.3. Confirmation of NGS-Assembled Genome

**Figure 3:**
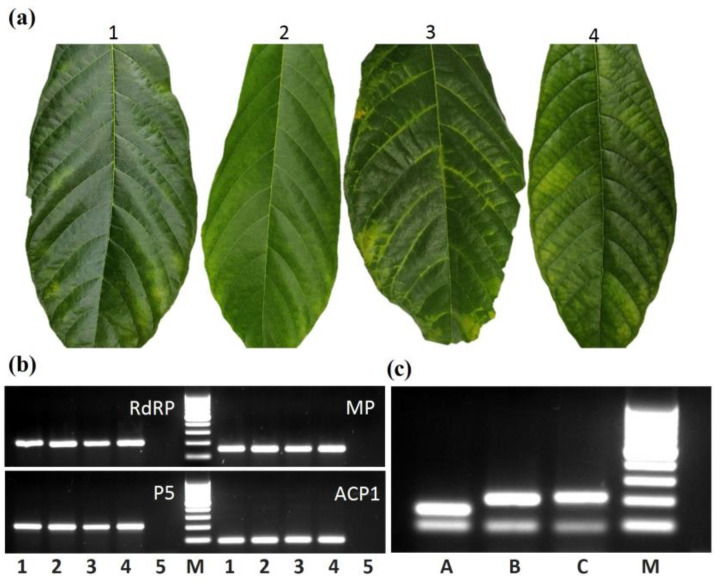
Detection of cacao polerovirus (CaPV). (**a**) Pictures of leaves collected from accessions ARF 2 (1), GU 139/A (2), RB 37 [BRA] (3) and TSH 1188 (4) for testing for CaPV. (**b**) RT-PCR-based detection of CaPV in cacao accessions 1–4, with primers designed from RNA-directed RNA polymerase (RdRP), movement protein (MP) and PLRV readthrough domain protein (P5). *T. cacao* acyl carrier protein 1 (ACP1) was used as internal control. Lane 5 was loaded with no-RT reaction. (**c**) Optimization of multiplex RT-PCR. Lanes A, B and C contained RT-PCR products amplified with a combination of ACP1 with RdRP, MP or P5 primers, respectively. A 100 bp increment ladder is shown in lanes M for size reference.

The near-complete NGS genome of CaPV was verified by RT-PCR amplification. The RNA-Seq libraries in the PSU study that we analysed were constructed from leaf samples obtained in 2018 from the cacao accessions held at CATIE. Hence, we decided to use the germplasm at ICQC-R, imported from CATIE, for the detection of CaPV. We initially selected three recently imported accessions, namely ARF 2, GU 139/A and TSH 1188, together with RB 37, which was imported in 2013 but exhibited prominent yellow vein symptoms (Figure 3a). The accessions were tested with three CaPV-specific primers sets, and a cacao-specific ACP1 primer set. All four primer sets amplified discrete bands of the expected sizes, of 150, 201, 212 and 90 bp, from CaPV genome regions encoding RdRP (P2), MP (P4) and readthrough domain proteins (P5), and the cacao ACP1 gene, respectively. No amplification was found in the No-Reverse Transcriptase (-RT) control (Figure 3b).

**Figure 4:**
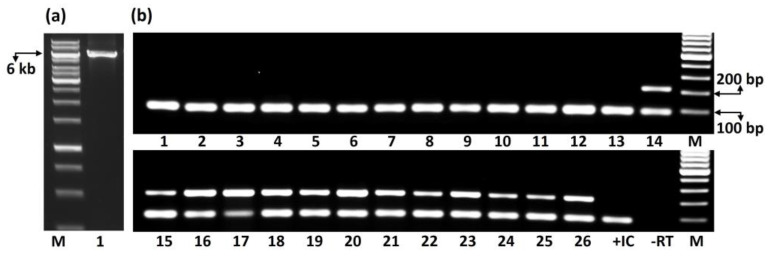
Agarose gel electrophoresis. (**a**) RT-PCR amplification of the complete genome of cacao Polerovirus virus (CaPV) from RB37. (**b**) Multiplex RT-PCR-based assay for testing of CaPV in the International Cocoa Quarantine Centre, Reading (ICQC-R) germplasm. A 212 bp fragment of CaPV and a 90 bp fragment of internal control [cacao ACP1 gene (LOC18599903)] were amplified from accessions RB 46 [BRA] (1), RB 49 [BRA] (2), RIM 189 [MEX] (3), EET 95 [ECU] (4), PMCT 93 (5), ARF 12 (6), PA 169 [PER] (7), CC 252 (8), CC 137 (9), EET 183 [ECU] (10), UF 712 (11), CRIOLLO 21 [CRI] (12), APA 5 (13), BE 8 (14), C SUL 3 (15), EET 387 [ECU] (16), BE 5 (17), RB 37 [BRA] (18), RB 43 [BRA] (19), RB 48 [BRA] (20), BE 2 (21), CAS 3 (22), CRIOLLO 17 [CRI] (23), GU 139/A (24), ARF 2 (25) and TSH 1188 (26). Letter M denotes the lanes loaded with 1 kb (**a**) or 100 bp size marker (**b**).

With regard to the amplification of the complete genome, primers designed from both ends of the NGS-derived CaPV genome failed to amplify the targeted sequence. A second set of primers designed from 5′ and 3′ UTRs, for amplification of the coding regions of CaPV, amplified a band of expected size (Figure 4a), which was cloned and sequenced by Sanger sequencing. Alignment of the Sanger contig of 5794 nt with the NGS-derived CaPV genome revealed > 99% identity with 98% coverage (nt 33–5826). No polymorphisms were found between both sequences in 5′, 3′ and intergenic UTRs. The ORF0, which encodes P0, contained six single nucleotide polymorphisms (SNPs). The P1 region encoded by ORF1 was found to be the most variable region with 26 SNPs. ORF2, which encodes RdRP, was found to be more conserved with seven SNPs. ORF3 and ORF4, which encode CP and MP, had one SNP each. Five SNPs were found in ORF5, which encodes the readthrough domain protein.

### 3.4. Screening of Germplasms

Singleplex diagnostic PCR assays are prone to false negative results, mainly due to the presence of PCR inhibitors in the reaction. We have developed multiplex RT-PCR assays that include a CaPV specific primer set and a cacao ACP1 primer set as ICs. The amplification of the ACP1 target would validate the negative results for CaPV. The accession RB 37 was used for the optimization of the multiplex assay. Combinations of ICs with each of the three CaPV primer sets successfully amplified the primary and secondary targets (Figure 3c).

Twenty-six cacao accessions, received from CATIE between 1998 and 2021, were tested with multiplex RT-PCR. Each accession was screened with three multiplex primer combinations. Figure 4b represents the RT-PCR products amplified with P5 and ACP1 primer combination and loaded on the gel in chronological order of accession date. Gel images of screening with two other primer combinations are presented in Figure S2. Thirteen accessions, imported from 1998 to 2011, tested negative, whereas the remaining 13 accessions, imported from 2011 to 2021, tested positive for CaPV (Figure 3b; Table S4). Pictures of the leaves of 13 accessions that tested positive did not show a consistent pattern of symptoms. (Figure S3). The accessions RB 37 [BRA], RB 43 [BRA] and RB 48 [BRA] showed yellow vein banding, whereas ARF 2 and TSH 1188 had interveinal yellowing. The remaining leaves did not show visible symptoms. Yellow vein banding was also observed in the leaf of RB 46, which tested negative for CaPV (Figure S4). It is noteworthy to mention that the set of 26 accessions tested for polerovirus tested negative for the presence of CaMMV and CYVBV.

## 4. Discussion

**Figure 5:**
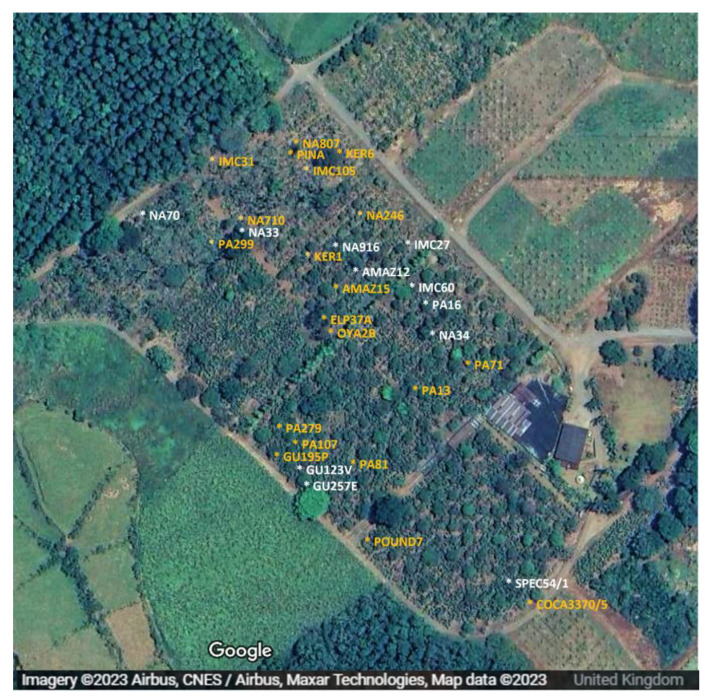
Location of the selected 31 cacao trees (BioProject PRJNA558793) held at Centro Agronómico Tropical de Investigación y Enseñanza (CATIE), Costa Rica. White and yellow font colour represent the trees tested positive and negative for Cacao polerovirus, respectively.

This study has generated several important findings. It has utilised the SRA transcript database to identify, for the first time, the widespread presence of a polerovirus, now named CaPV, in a range of cacao germplasms, and to reconstruct a near-complete genome with all the genome features known for poleroviruses from other species [14]; previously, only a fragment of the genome had been reported in cacao [16]. The full-length sequence was then used to design primers that enabled the cloning of a genomic sequence and then the development of diagnostic primers, which were used to screen a set of germplasms in the ICQC-R collection. This analysis identified virus-positive plants from a range of diverse germplasms and confirmed that these plants all originated from material transferred from CATIE to the ICQC-R between 2011 and 2021. The CATIE germplasm was also the source of the leaves collected in 2018 by PSU and found to give the positive results in the SRA database (Table 2). It is important to note that not all the leaves from the PSU study tested positive (Figure 5), and that there is no suggestion that all cacao material at CATIE is infected. In addition, the data show infected trees from several genetic groups (Table 2), a finding that suggests there is no obvious genetic basis for the presence or absence of infection.

These results can now be used to extend the understanding of viruses known to infect cacao and, also, to inform policy relating to the international transfer of cacao germplasms. As regards the diversity of the cacao virome, previous studies have focused almost exclusively on badnaviruses, particularly on those known to cause CSSVD, but also on the mild strains of CaMMV and CYVBV. The latter viruses are known to occur in asymptomatic plants [7]. In this context, it is especially relevant now to consider the evidence for any link between the presence of the CaPV and symptomology. On one hand, the previous studies [16,17] to identify the presence of a polerovirus in cacao were conducted on plants that showed symptoms but did not find the virus in all symptomatic plants [17]. On the other hand, some asymptomatic plants tested positive in the present study (Figure 3a; Figure S3). Moreover, there is a broad range of reported symptoms related to infections caused by cacao mild badnaviruses [4,5,6,8], and, in some cases, these symptoms are similar to those reported for polerovirus infection [17]. Taken together, these results provide no certainty about any potential impact of CaPV on cacao production, and no information on any possible synergy between CaPV and other viruses, nor on whether the symptoms may be exacerbated by nutrient deficiency, known to cause similar symptoms in leaves [32]. These issues can only be resolved by a much more thorough large-scale analysis of plants deliberately co-infected with CaPV and mild strains of badnaviruses, and their possible interactions with abiotic stresses.

The present findings also provide some limited circumstantial evidence concerning the origin of the CaPV in the CATIE collection. As shown in Table S4, virus-positive plants were limited to those received in the ICQC-R after 2010; those received prior to that date tested negative. Possible explanations for this variation include latent infection in germplasms introduced into the CATIE gene bank in the 2000s, cross infection during repropagation, or a sudden infection of the collection, from an unknown source, prior to 2010. Interestingly, there was no evidence of any spatial clustering in the infected clones in the field (Figure 5), a finding that might argue against the infection having entered the field from any specific direction. It would be informative to conduct a study of plants growing close to the collection, which might act as alternative host for CaPV and as a reservoir of infection. For example, it has been reported that another polerovirus, cotton leafroll dwarf virus, was found in 23 species of weeds growing adjacent to cotton fields in Georgia, USA [33]. A study of insects in the vicinity of the CATIE collection would also be valuable. It is reported that poleroviruses, such as CLRDV [34], are only, or mostly, transmitted by the cotton aphid *Aphis gossypii*, though there is no available experimental evidence of this from cacao. However, there is some evidence that biolistic methods can be used for the mechanical transmission of other species of poleroviruses [34], and it would be helpful to test the possibility of mechanical transmission of CaPV.

In summary, this study has extended the diversity of viruses known to infect cacao and provided robust diagnostic technique for CaPV. It should be noted that such discovery is an ongoing process and, as shown in other crops, it is likely that the virome will be extended, potentially to many more species. For example, five new poleroviruses were recently identified by detailed analysis of transcriptome data from six plant species [35]. At a practical level, the data provided here confirm the value of molecular diagnostic testing in complementing virus-indexing by visual observation, to ensure the safe international movement of cacao germplasms.

## Figures and Tables

**Table 1:** Primers used in cloning and detection of cacao polerovirus. RdRP, MP and P5 denote RNA-directed RNA polymerase, movement protein and readthrough domain protein, respectively. ACP1 denotes *Theobroma cacao* acyl carrier protein 1.

Specificity	Primer Location	Primer Name	Primer Sequence	Coordinates (nt)	Amplicon Size (bp)
Cacao polerovirus	5′ UTR	CLN_F	CGACCGTATCATTGCTTGTG	33–52	5794
	3′ UTR	CLN_R	CCATGCCTGTGGGTTTTAAG	5807–5826	
	RdRP (ORF2)	P2_F	GTACCCTCCTAGCCCAGACC	3106–3125	150
		P2_R	CTGGGGATTCTAAGGCATCA	3236–3255	
	MP (ORF4)	P4_F	AGGGAGCAACAGCTTCACA	3954–3972	201
		P4_R	GAGATGGAGCCTGATGTCGT	4135–4154	
	P5 (ORF5)	P5_F	CAAGGAGTGCTGGTCGTACA	4990–5009	212
		P5_R	GGGGTTTGATTTACCGGTTT	5182–5203	
*Theobroma cacao*	ACP1_Exon2	ACP_F	GAAGCAGCCTCTCATTTAATTTG	1148–1170	* 90
	ACP1_Exon3	ACP_R	CACATACCTTATCCACAGTCTCT	1754–1776	** 629

* Size from mRNA (XM_007030091.2); ** Size from gene (LOC18599903).

**Table 2:** List of the RNA-Seq datasets in BioProject PRJNA558793 that contained a significant number of reads mapped to partial cacao polerovirus sequences. Regular and italic font styles represent accessions held at Centro Agronómico Tropical de Investigación y Enseñanza (CATIE), Costa Rica, and Pennsylvania State University (PSU), USA, respectively.

Run	Station/GPS Coordinates	Accession	Genetic Group	Size (Mb)	Reads Mapped	Coverage (X)
SRR9938230	CATIE/9.87675, −83.65673333	IMC 60	Iquitos	1008	153	14.1
*SRR23566402*	*Experimental Greenhouse J, PSU*	*IMC60_S62*		*544*	*93*	*13.8*
SRR9938245	CATIE/9.876666667, −83.65666667	PA 16	Maranon	845	135	12.0
*SRR23566388*	*Experimental Greenhouse J, PSU*	*PA16_S70*		*456*	*75*	*13.9*
SRR9938323	CATIE/9.876466667, −83.65663333	NA 34	Iquitos	1010	64	5.7
*SRR23566397*	*Experimental Greenhouse J, PSU*	*NA34_S80*		*548*	*39*	*6.4*
SRR9938250	CATIE/9.87575, −83.65738333	GU 123/V	Guiana	1168	54	5.0
*SRR23566375*	*Experimental Greenhouse J, PSU*	*GU123V_S57*		*634*	*39*	*5.1*
SRR9938307	CATIE/9.8771, −83.65805	NA 70	Nanay	990	51	4.6
*SRR23566396*	*Experimental Greenhouse J, PSU*	*NA70_S81*		*538*	*37*	*5.7*
SRR9938308	CATIE/9.877016667, −83.6576	NA 33	Nanay	2443	51	4.4
*SRR23566398*	*Experimental Greenhouse J, PSU*	*NA33_S79*		*1290*	*21*	*4.2*
SRR9938223	CATIE/9.8757, −83.65738333	GU 257/E	Guiana	1068	41	3.6
*SRR23566373*	*Experimental Greenhouse J, PSU*	*GU257E_S52*		*576*	*31*	*4.5*
SRR9938227	CATIE/9.875166667, −83.65623333	SPEC 54/1	Iquitos	1099	32	2.9
*SRR23566379*	*Experimental Greenhouse J, PSU*	*SPEC54/1_S63*		*596*	*28*	*3.2*
SRR9938231	CATIE/9.876783333, −83.65706667	AMAZ 12	Iquitos	1139	30	2.8
*SRR23566405*	*Experimental Greenhouse J, PSU*	*AMAZ12_S64*		*613*	*9*	*1.2*
SRR9938234	CATIE/9.8769, −83.65676667	IMC 57	Iquitos	1016	28	2.5
*SRR23566403*	*Experimental Greenhouse J, PSU*	*IMC57_S67*		*522*	*7*	*0.8*
SRR9938218	CATIE/9.87695, −83.65716667	NA 916	Nanay	955	26	2.4
*SRR23566391*	*Experimental Greenhouse J, PSU*	*NA916_S77*		*518*	*20*	*2.4*

## Data Availability

Sequencing data generated in this study were submitted to NCBI GenBank and have accession numbers OR605721 and Third Party Annotation (TPA) database accession number BK064704.

## Supplementary Materials

The following supporting information can be downloaded at: https://www.mdpi.com/article/10.3390/pathogens12111284/s1. Figure S1: Multiple sequence alignment of the de novo assembled cacao polerovirus contig (CaPV) discovered in this study and the previously reported five partial sequences of cacao leafroll virus; Figure S2: agarose gel electrophoresis; Figure S3: pictures of sampled leaves from the accessions that tested positive for cacao polerovirus; Figure S4: pictures of sampled leaves from the accessions that tested negative for cacao polerovirus; Table S1: summary of cacao (*Theobroma cacao* L.) RNA sequencing datasets used in the study; Table S2: sequence identity of the cacao polerovirus isolate discovered in this study with 43 selected species from four genera of the *Solemoviridae* family using amino acid sequences of the coat protein; Table S3: sequence identity of the cacao polerovirus isolate discovered in this study with 43 selected species from four genera of *Solemoviridae* family using amino acid sequences of RNA-directed RNA polymerase; Table S4: summary of multiplex RT-PCR test for the detection of cacao polerovirus in selected germplasms held at International Cocoa Quarantine Centre, Reading, UK (ICQC-R).
