# Supplementary material for: Identification of a Novel Polerovirus in Cocoa (Theobroma cacao) Germplasm and Development of Molecular Methods for Use in Diagnostics
Source: Pathogens. 2023 Oct 26;12(11):1284. doi: 10.3390/pathogens12111284 (PMC10674516; doi:10.3390/pathogens12111284)
Supplement: Supplementary file 1 [file pathogens-12-01284-s001.zip › pathogens-2684931-supplementary.pdf]

Identification of a novel polerovirus in cocoa (*Theobroma cacao*) germplasm and development of molecular methods for use in diagnostics.

Ihsan Ullah, Muhammad Kamran and Jim M. Dunwell

Supplementary Figures

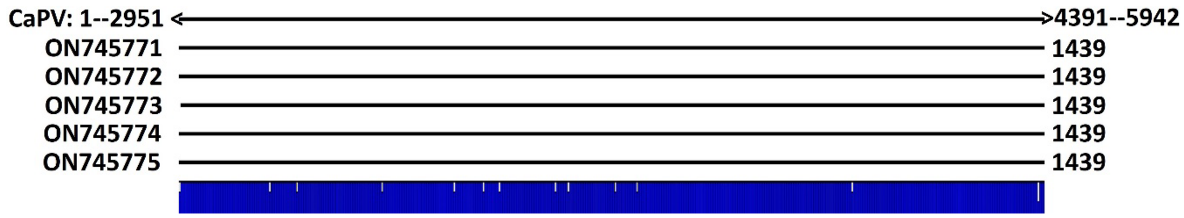

Figure S1. Multiple sequence alignment of the de novo assembled cacao polerovirus contig (CaPV) discovered in this study and the previously reported five partial sequences of cacao leafroll virus.

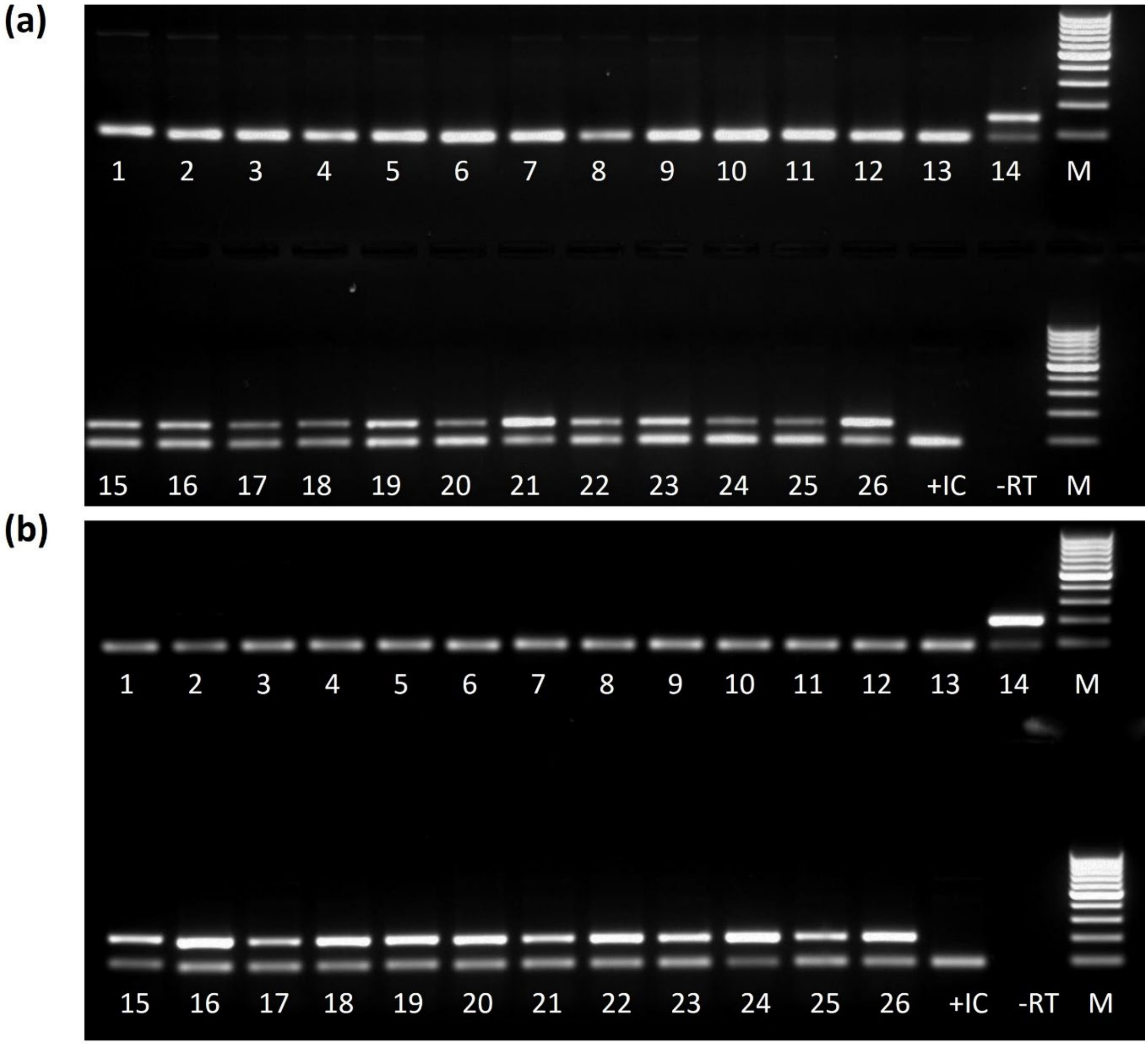

Figure S2. Agarose gel electrophoresis. Multiplex RT-PCR-based assay for testing of CaPV in the International Cocoa Quarantine Centre, Reading (ICQC-R) germplasm. Using P1 (a) or MP (b) virus specific primer set and a 90 bp fragment of internal control (cacao ACP1 gene (LOC18599903)).Loading sequencing is as follows. RB 46 [BRA] (1), RB 49 [BRA] (2), RIM 189 [MEX] (3), EET 95 [ECU] (4), PMCT 93 (5), ARF 12 (6), PA 169 [PER] (7), CC 252 (8), CC 137 (9), EET 183 [ECU] (10), UF 712 (11), CRIOLLO 21 [CRI] (12), APA 5 (13), BE 8 (14), C SUL 3 (15), EET 387 [ECU] (16), BE 5 (17), RB 37 [BRA] (18), RB 43 [BRA] (19), RB 48 [BRA] (20), BE 2 (21), CAS 3 (22), CRIOLLO 17 [CRI] (23), GU 139/A (24), ARF 2 (25) and TSH 1188 (26).. Letter M denotes the lanes loaded with 100 bp size marker.

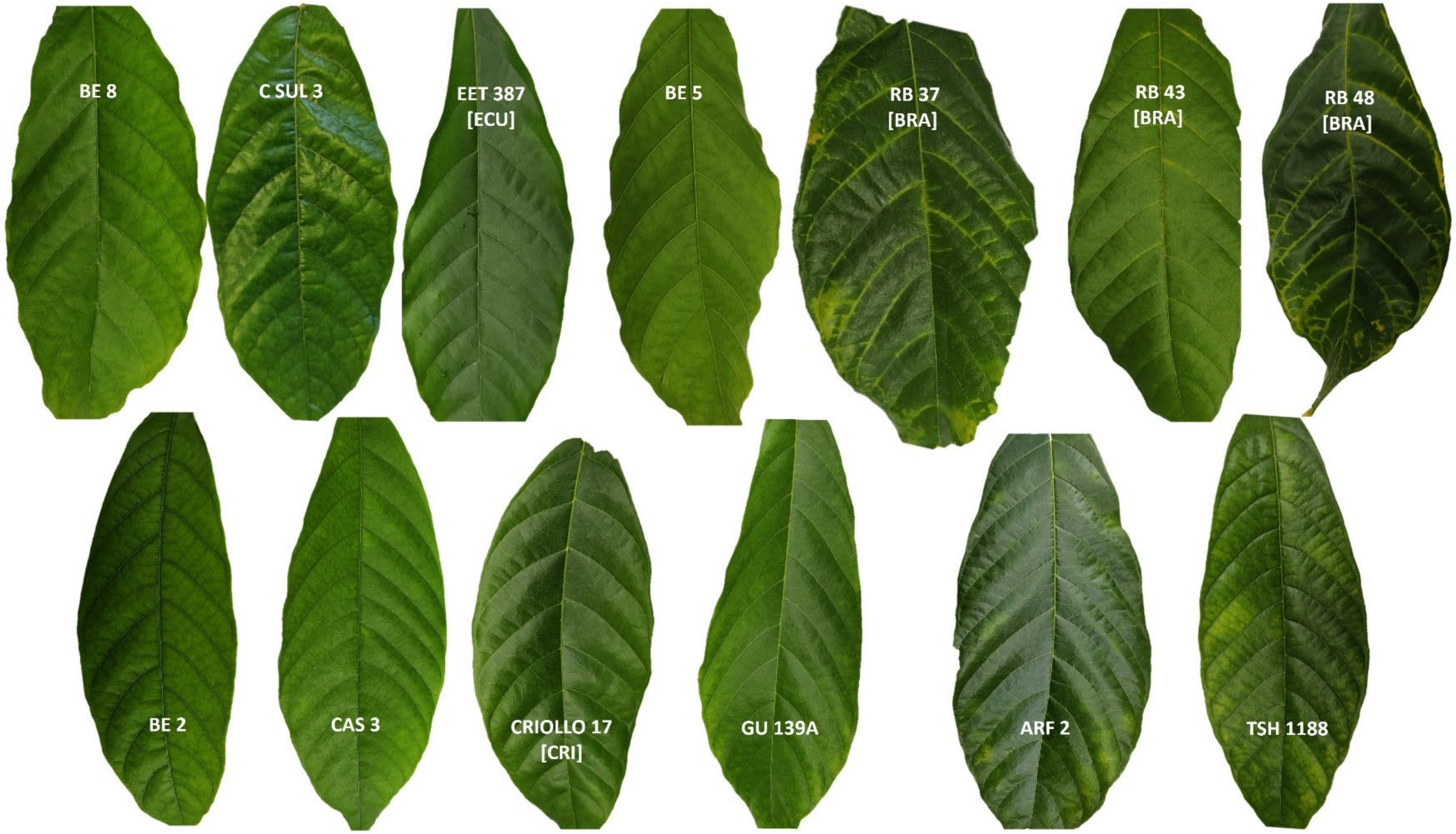

Figure S3. Pictures of sampled leaves from the accessions that tested positive for cacao polerovirus. The accessions were imported from Centro Agronómico Tropical de Investigación y Enseñanza (CATIE), Costa Rica, between 2011 and 2021, and are held at International Cocoa Quarantine Centre, Reading, UK (ICQC-R).

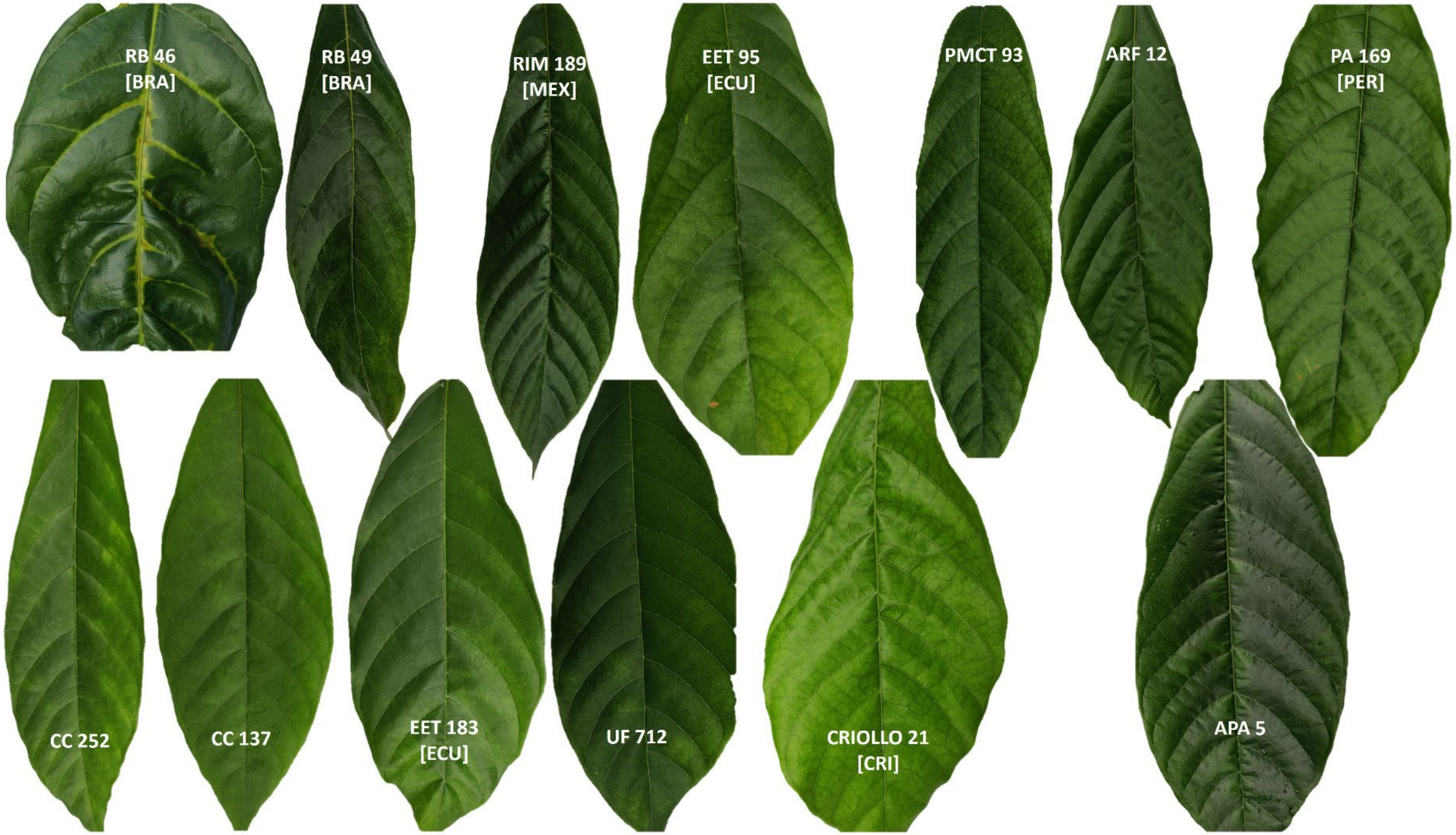

Figure S4. Pictures of sampled leaves from the accessions that tested negative for cacao polerovirus. The accessions were imported from Centro Agronómico Tropical de Investigación y Enseñanza (CATIE), Costa Rica, between 1988 and 2011, and are held at International Cocoa Quarantine Centre, Reading, UK (ICQC-R).

Supplementary tables

Table S1. Summary of cacao (*Theobroma cacao* L) RNA sequencing datasets used in the study.

BioProject	Number of SRA experiments	Read length	Data volume (G bases)	Number of cacao accessions in the study	Year of data release
PRJNA971242	42	300	271	6	2023
PRJNA933172	388	75	267	3	2023
PRJNA931994	30	101	16	3	2023
PRJEB35419	12	300	106	2	2023
PRJNA785999	13	300	87	3	2022
PRJNA742476	12	100	24	1	2021
PRJNA714293	3	297	116	1	2021
PRJNA613342	9	300	61	3	2020
PRJNA604260	3	300	24	1	2020
PRJNA558793	272	75–281	562	40	2019, 2023
PRJNA476877	72	100	144	2	2018
PRJNA471714	36	100	73	1	2018
PRJNA421343	3	300	151	1	2018
PRJNA413468	1	50	0.4	1	2017
PRJNA326055	10	200	32	10	2019
PRJNA314774	20	200	60	20	2016
PRJNA189464	10	96	96	1	2014–2015
PRJNA51633	18	108–548	88	1	2015

Table S2. Sequence identity of cacao polerovirus isolate discovered in this study with 43 selected species from four genera of the *Solemoviridae* family using amino acid sequences of the coat protein.

Accession	Species	Genus	Percent identity (%)
YP_003915151	Cotton leafroll dwarf virus	Polerovirus	57
YP_001949873	Melon aphid borne yellows virus	Polerovirus	57
NP_ 840023	Cereal yellow dwarf virus RPV	Polerovirus	56
YP_010086858	Faba bean polerovirus 1	Polerovirus	56
NP_ 612216	Beet mild yellowing virus	Polerovirus	55
NP_ 840099	Beet western yellows virus	Polerovirus	55
NP_ 054688	Cereal yellow dwarf virus RPS	Polerovirus	55
YP_667841	Chickpea chlorotic stunt virus	Polerovirus	55
NP_ 620104	Cucurbit aphid borne yellows virus	Polerovirus	55
YP_009506760	Groundnut rosette assistor virus	Polerovirus	55
YP_006666509	Suakwa aphid borne yellows virus	Polerovirus	55
NP_ 620488	Turnip yellows virus	Polerovirus	55
NP_ 114362	Beet chlorosis virus	Polerovirus	54
YP_009254742	Pepo aphid borne yellows virus	Polerovirus	54
NP_ 056749	Potato leafroll virus	Polerovirus	53
YP_010087207	Pumpkin polerovirus	Polerovirus	53
YP_008083742	Maize yellow dwarf virus RMV	Polerovirus	50
YP_077189	Carrot red leaf virus	Polerovirus	49
YP_001931933	Tobacco vein distorting virus	Polerovirus	49
YP_010782936	Maize yellow mosaic virus	Polerovirus	49
YP_009455744	Pepper vein yellows virus 5	Polerovirus	49
NP_ 050008	Sugarcane yellow leaf virus	Polerovirus	40
YP_009373266	Grapevine enamovirus 1	Enamovirus	38
YP_009249826	Alfalfa enamovirus 1	Enamovirus	32
YP_004869652	Soybean yellow common mosaic virus	Sobemovirus	29
YP_009344993	Solanum nodiflorum mottle virus	Sobemovirus	28
NP_ 941377	Cocksfoot mottle virus	Sobemovirus	26
YP_002308437	Imperata yellow mottle virus	Sobemovirus	26
YP_007438859	Southern bean mosaic virus	Sobemovirus	26
YP_009140474	Cymbidium chlorotic mosaic virus	Sobemovirus	25
YP_010087763	Physalis rugose mosaic virus	Sobemovirus	25
YP_002308463	Poinsettia latent virus	Polemovirus	25
YP_009142786	Rottboellia yellow mottle virus	Sobemovirus	25
YP_007438863	Rice yellow mottle virus	Sobemovirus	24
YP_007438851	Lucerne transient streak virus	Sobemovirus	23
YP_008869288	Turnip rosette virus	Sobemovirus	23
YP_003896040	Velvet tobacco mottle virus	Sobemovirus	23
YP_002158815	Sowbane mosaic virus	Sobemovirus	22
YP_006331063	Artemisia virus A	Sobemovirus	21
YP_006589927	Papaya lethal yellowing virus	Sobemovirus	21
YP_007438855	Ryegrass mottle virus	Sobemovirus	21
NP_ 715629	Subterranean clover mottle virus	Sobemovirus	21
NP_ 042303	Southern cowpea mosaic virus	Sobemovirus	18

Table S3. Sequence identity of cacao polerovirus isolate discovered in this study with 43 selected species from four genera of *Solemoviridae* family using amino acid sequences of RNA-directed RNA polymerase.

Accession	Species	Genus	Percent identity (%)
YP 002308462	Poinsettia latent virus	Polemovirus	62
NP 050007	Sugarcane yellow leaf virus	Polerovirus	58
NP 620479	Beet mild yellowing virus	Polerovirus	50
NP 620485	Turnip yellows virus	Polerovirus	49
NP 056748	Potato leafroll virus	Polerovirus	49
YP 077186	Carrot red leaf virus	Polerovirus	48
YP 010800294	Groundnut rosette assistor virus	Polerovirus	48
NP 620101	Cucurbit aphid borne yellows virus	Polerovirus	48
YP 003915148	Cotton leafroll dwarf virus	Polerovirus	48
YP 009455740	Pepper vein yellows virus 5	Polerovirus	48
YP 008083739	Maize yellow dwarf virus RMV	Polerovirus	48
YP 010086855	Faba bean polerovirus 1	Polerovirus	47
YP 010782933	Maize yellow mosaic virus	Polerovirus	47
YP 001949870	Melon aphid borne yellows virus	Polerovirus	47
NP 840097	Beet western yellows virus	Polerovirus	47
NP 840022	Cereal yellow dwarf virus RPV	Polerovirus	47
NP 054685	Cereal yellow dwarf virus RPS	Polerovirus	46
YP 667838	Chickpea chlorotic stunt virus	Polerovirus	46
YP 001931931	Tobacco vein distorting virus	Polerovirus	45
YP 009254738	Pepo aphid borne yellows virus	Polerovirus	45
NP 114361	Beet chlorosis virus	Polerovirus	44
YP 010087203	Pumpkin polerovirus	Polerovirus	43
YP 006666506	Suakwa aphid borne yellows virus	Polerovirus	43
NP 736581	Subterranean clover mottle virus	Sobemovirus	39
YP 010087762	Physalis rugose mosaic virus	Sobemovirus	39
NP 942020	Cocksfoot mottle virus	Sobemovirus	37
YP 010790432	Grapevine enamovirus 1	Enamovirus	34
YP 009249823	Alfalfa enamovirus 1	Enamovirus	33
YP 009142784	Rottboellia yellow mottle virus	Sobemovirus	32
YP 008869286	Turnip rosette virus	Sobemovirus	32
YP 006589925	Papaya lethal yellowing virus	Sobemovirus	32
YP 009140472	Cymbidium chlorotic mosaic virus	Sobemovirus	31
YP 009344991	Solanum nodiflorum mottle virus	Sobemovirus	31
YP 003896039	Velvet tobacco mottle virus	Sobemovirus	31
YP 007438858	Southern bean mosaic virus	Sobemovirus	31
NP 042302	Southern cowpea mosaic virus	Sobemovirus	31
YP 002158813	Sowbane mosaic virus	Sobemovirus	30
YP 004869651	Soybean yellow common mosaic virus	Sobemovirus	30
YP 007438853	Ryegrass mottle virus	Sobemovirus	30
YP 007500964	Rice yellow mottle virus	Sobemovirus	29
YP 007438849	Lucerne transient streak virus	Sobemovirus	29
YP 006331061	Artemisia virus A	Sobemovirus	28
YP 007506946	Imperata yellow mottle virus	Sobemovirus	28

Table S4. Summary of multiplex RT-PCR test for the detection of cacao polerovirus in selected germplasm, held at International Cocoa Quarantine Centre, Reading, UK (ICQC-R). The accessions were imported from Centro Agronómico Tropical de Investigación y Enseñanza (CATIE), Costa Rica, between 1988 and 2021.

Serial number	Accession name	ICQC Number	Import year	Test result
1	RB 46 [BRA]	RUQ 0134	1988	-ve
2	RB 49 [BRA]	RUQ 0136	1988	-ve
3	RIM 189 [MEX]	RUQ 0310	1992	-ve
4	EET 95 [ECU]	RUQ 0414	1993	-ve
5	PMCT 93	RUQ 1249	2002	-ve
6	ARF 12	RUQ 1364	2003	-ve
7	PA 169 [PER]	RUQ 1479	2004	-ve
8	CC 252	RUQ 1506	2004	-ve
9	CC 137	RUQ 1637	2007	-ve
10	EET 183 [ECU]	RUQ 1640	2007	-ve
11	UF 712	RUQ 1683	2009	-ve
12	CRIOLLO 21 [CRI]	RUQ 1682	2009	-ve
13	APA 5	RUQ 1695	2011	-ve
14	BE 8	RUQ 1694	2011	+ve
15	C SUL 3	RUQ 1704	2013	+ve
16	EET 387 [ECU]	RUQ 1703	2013	+ve
17	BE 5	RUQ 1719	2013	+ve
18	RB 37 [BRA]	RUQ 1705	2013	+ve
19	RB 43 [BRA]	RUQ 1717	2013	+ve
20	RB 48 [BRA]	RUQ 1720	2013	+ve
21	BE 2	RUQ 1733	2015	+ve
22	CAS 3	RUQ 1744	2016	+ve
23	CRIOLLO 17 [CRI]	RUQ 1742	2016	+ve
24	GU 139 /A	RUQ 1760	2020	+ve
25	ARF 2	RUQ 1762	2021	+ve
26	TSH 1188	RUQ 1761	2021	+ve
